# Pre-trained convolutional neural networks identify Parkinson’s disease from spectrogram images of voice samples

**DOI:** 10.1038/s41598-025-92105-6

**Published:** 2025-03-01

**Authors:** Yasir Rahmatallah, Aaron S. Kemp, Anu Iyer, Lakshmi Pillai, Linda J. Larson-Prior, Tuhin Virmani, Fred Prior

**Affiliations:** 1 Biomedical Informatics, University of Arkansas for Medical Sciences, Little Rock, 72205 USA; 2 Georgia Institute of Technology, Atlanta, 30332 USA; 3 Neurology, University of Arkansas for Medical Sciences, Little Rock, 72205 USA; 4 Neuroscience, University of Arkansas for Medical Sciences, Little Rock, 72205 USA

**Keywords:** Parkinson's disease, Learning algorithms, Parkinson's disease

## Abstract

Machine learning approaches including deep learning models have shown promising performance in the automatic detection of Parkinson’s disease. These approaches rely on different types of data with voice recordings being the most used due to the convenient and non-invasive nature of data acquisition. Our group has successfully developed a novel approach that uses convolutional neural network with transfer learning to analyze spectrogram images of the sustained vowel /a/ to identify people with Parkinson’s disease. We tested this approach by collecting a dataset of voice recordings via analog telephone lines, which support limited bandwidth. The convolutional neural network with transfer learning approach showed superior performance against conventional machine learning methods that collapse measurements across time to generate feature vectors. This study builds upon our prior results and presents two novel contributions: First, we tested the performance of our approach on a larger voice dataset recorded using smartphones with wide bandwidth. Our results show comparable performance between two datasets generated using different recording platforms despite the differences in most important features resulting from the limited bandwidth of analog telephonic lines. Second, we compared the classification performance achieved using linear-scale and mel-scale spectrogram images and showed a small but statistically significant gain using mel-scale spectrograms.

**Supplementary Information:**

The online version contains supplementary material available at 10.1038/s41598-025-92105-6.

## Introduction

Clinical diagnosis of Parkinson’s disease (PD) is based on motor symptoms defined by bradykinesia plus one or more of an additional 3 features that include rigidity, rest tremor and postural instability^1,2^. In addition to disturbances of posture and gait, speech abnormalities are found in up to 90% of people with PD (PwPD) as reported in a large body of literature^3–7^. The use of machine learning approaches for the automatic classification of PwPD and healthy controls (HC) from voice samples has grown over the past decade. Typically, sustained vowel phonation is used to evaluate phonation features, while connected speech has been used to evaluate articulatory and prosodic features^8–10^. Recent advances in deep learning and transfer learning (pre-trained) models with convolutional neural networks (CNNs) have led to a renewed interest in spectrogram images of voice to perform different tasks, including identification of PwPD. Spectrograms are two-dimensional presentations that show a signal’s energy distribution across time and frequency. Recent studies have demonstrated success in using spectrograms to distinguish PwPD and HC. Hires et al.^8^ used an ensemble of CNNs to detect PwPD in spectrogram images of vowel sounds from 50 PwPD and 50 HC. This approach adopted the Xception^11^ model trained on ImageNet^12^ to generate image features, with the model fine-tuned using two datasets separately: A dataset of vowels^13^ and the Saarbruecken Voice Database (SVD)^14^ of speech recordings. The best performance was achieved with the sustained vowel /a/ (AUC = 0.89) in recordings from the PC-GITA^15^ dataset acquired under a controlled environment (recorded using the same device with supervision in the same quiet room). Vasquez-Correa et al.^16^ provided a deep learning approach for discriminating 44 PwPD and 39 HC based on analysis of a multimodal dataset consisting of handwriting, gait, and speech tasks. Using speech tasks alone, they achieved 0.92 ~ 0.96 Area Under the receiver operating characteristic Curve (AUC), by analyzing spectrograms of the transitions between unvoiced to voiced speech segments. Worasawate et al.^17^ used CNN models to distinguish PwPD and HC from spectrograms of the sustained vowel /a/ uttered by 523 PwPD and 3,528 HC participants over 35 years of age from the mPower dataset^18^. Each recording was sliced into 1-second segment which was converted into a spectrogram image, resulting in 9,929 and 19,869 spectrograms for PwPD and HC, respectively. Although the best CNN model achieved about 99% accuracy, it is worth noting that two factors may have contributed to an over-optimistic classification rate in this study: First, the age of participants in the mPower dataset is severely skewed towards older age in the PwPD group and younger age in the HC group which leads to bias when a large number of participants is included without applying a more stringent age range criterion. Second, having multiple spectrograms generated from the same participant leads to identity confounding where these spectrograms show up in both the training and testing sets of the dataset, potentially leading the model to capture information associated with individuals rather than with the PD status. Additional studies have used CNN-based approaches to distinguish PwPD and HC from spectrogram images^19,20^. CNNs with transfer learning models have also been used to identify PwPD and HC from mel-scale spectrogram images of sustained vowels or continuous speech^21–27^. The mel-frequency scale models the perceptual frequency response of the human ear which is approximately linear below 1 kHz and nonlinear (logarithmic) above 1 kHz. The relationship between mel and Hz frequency scales is given by $\:{freq}_{mel}=2595\times\:{\text{log}}_{10}\left(1+{freq}_{Hz}/700\right)$. The mel-scale has been applied to classic voice feature vectors such as cepstral coefficients to generate mel-frequency cepstral coefficients and has shown good performance in speech applications, including the ability to detect voice disorders^28^, depression^29^, amyotrophic lateral sclerosis^30^, and PD^31–35^. While some studies claim that mel-scale spectrogram offers advantage over the linear-scale spectrogram in different tasks^22,36^, empirical results substantiating this claim in the context of distinguishing PwPD from HC remain scarce in literature.

Most of the studies available in the literature were conducted using voice recorded using professional grade microphones under controlled settings and high bandwidth (e.g. 16–44.1 kHz sampling frequency) with only few studies exploring the use of recordings captured using telephones under uncontrolled settings^37–40^, that is participants are in different environments and with differing levels of ambient noise when voice is recorded. There is also an important distinction that needs to be made between recordings captured using smartphones and transferred digitally using a software application, compared to voice samples captured using any type of phone, transferred in real time via analog telephonic lines, and recorded using digital voicemail (voice messages). Telephonic lines support a limited bandwidth (0.3 ~ 3.4 kHz) thereby affecting voice quality. However, older adults who traditionally are thought to struggle with technology, may find it easier to make a direct call and leave a voice message. One study found correlation between voice features of recordings captured by both professional microphones and smartphone microphones and deemed both reliable in detecting pathological voice in clinical settings^41^. However, other studies found poor generalizability when using specific features across datasets collected under different environments. For example, Carron et al.^38^ analyzed the impact of uncontrolled and unsupervised settings on the classification of 30 PwPD and 30 HC using the sustained vowel /a/ from recordings captured using a smartphone under controlled settings (same room and supervised) compared to a subset of similar size from the mPower dataset^18^ recorded using a smartphone under uncontrolled settings (different places and unsupervised). The study achieved good performance in classifying PwPD and HC in each dataset where the best classifier achieved an average AUC of 0.97 using the dataset collected during the study and 0.75 using the subset from the mPower dataset. However, the classifier failed when one dataset was used for training and another for testing. This result is expected since the study showed that the best features differentiating PwPD and HC were different between the two datasets. Pah et al.^42^ reported a similar pattern where features associated with vocal folds vibration performed well in classifying PwPD and HC using a dataset captured by smartphone in a noise-restricted room but performed poorly using the PC-GITA dataset^15^ captured by a professional grade microphone under controlled clinical settings. These contradictory results cast doubt on the applicability of a specific method and/or feature set across different recording platforms. However, our group has also recently shown the reliability of voice recordings collected via analog telephone lines under uncontrolled settings to classify PwPD and HC^40^ using a CNN with transfer learning applied to spectrograms generated from these telephonic recordings. In either case, both smartphone applications and digital voicemail options allow wide access to participants in different populations especially in rural and medically underserved areas^43^.

Our current study builds upon our prior results and presents two novel contributions: First, we show the reliability of the convolutional neural network with transfer learning (Inception V3 model) approach proposed earlier by our group^40^, and shown to perform well in identifying PwPD in recordings captured in real time and transferred via analog telephonic lines, when applied to a relatively larger dataset recorded using smartphones with a wider bandwidth and transferred digitally. Our results show that the limited bandwidth of the analog telephonic lines which causes attenuation to low frequency bands including the fundamental frequency is not detrimental to the classification performance. Second, we compare the classification performance achieved using our approach with linear-scale and mel-scale spectrogram images and show a small but statistically significant gain when using mel-scale spectrograms. Our results provide empirical evidence supporting the adoption of mel-scale spectrograms in the context of classifying PwPD and HC from the sustained vowel /a/.

## Results

### Study populations

Voice samples of the sustained vowel /a/ from two datasets were used in this study. The UAMS dataset was collected from PwPD and HC study participants by leaving a recorded voice message via telephone lines as previously described^40^. The dataset is publicly available and consists of voice recordings from 40 PwPD and 41 HC. The mPower dataset^18^ available from the Synapse database^44^, was collected using the mPower app on iPhones. We used mPower recordings from a sub-population that met the following criteria: (1) Age between 50 and 70 year-old to be more consistent with older adults who are diagnosed with PD, (2) participants who did not report conditions that affect voice, (3) PwPD who reported recording their voice before or at any time except just after taking their PD medications, since PD medications such as Levodopa can affect voice quality, and (4) the recordings had no excessive noise or interfering sounds when one author (YR) listened to them. The filtering criteria resulted in 188 PwPD and 210 HC recordings from 215 PwPD and 229 HC initially selected subjects, referred to simply as the mPower dataset hereafter. Table 1 provides demographics of the participants in the UAMS dataset and the selected sub-population of the mPower dataset.

**Table 1:** Demographics of participants considered in this study from the UAMS and mPower datasets.

	UAMS dataset		mPower dataset	
	Healthy controls (*n* = 41)	Parkinson’s disease (*n* = 40)	Healthy controls (*n* = 210)	Parkinson’s disease (*n* = 188)
Sex (male/female)	16/25	21/19	174/36	121/67
Age at enrollment (years)	47.9 ± 14.5	66.6 ± 9.0	57.6 ± 5.6	61.1 ± 5.4

### Classification results using acoustic features

**Fig. 1:**
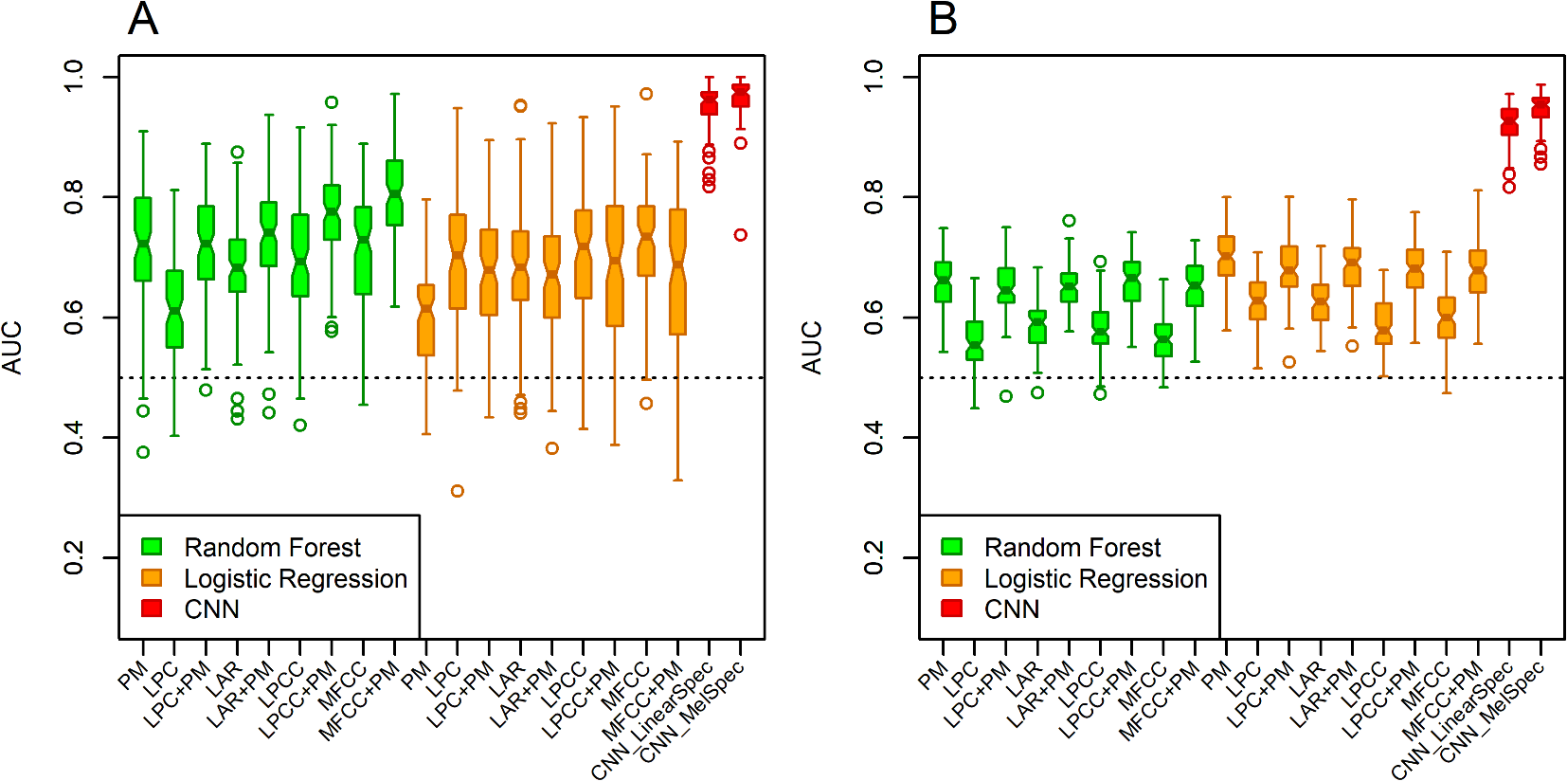
Estimated classification performance metric quantified by the area under the receiver operating characteristic curve (*AUC*) achieved in 100 iterations using random forest (*RF*) and logistic regression (*LR*) classifiers with the Parselmouth (*PM*) and variance feature vectors of four types of spectral features (separately and combined) and using the pre-trained convolutional neural network (*CNN*) with mel-scale and linear-scale spectrogram images. (**A**) Results from the UAMS dataset, (**B**) results from the mPower dataset. *LPC:* linear prediction coding, *LAR:* log area ratio, *LPCC:* linear prediction cepstral coefficients, *MFCC:* mel-frequency cepstral coefficients.

A feature vector of 23 traditionally studied features related to phonation in sustained vowels was estimated using Parselmouth^45^ for each of the recordings from PwPD and HC. These features were selected based on their frequent use in the literature. Logistic regression (LR) and random forest (RF) classifiers were applied to estimate classification performance from feature vectors (see the Methods for details) in 100 iterations. For the UAMS dataset, the RF classifier outperformed the adaptive LR model, achieving an average AUC of 0.72 against 0.6 for LR. For the mPower dataset, an opposite trend was observed with smaller differences (Table 2; Fig. 1). It is worth stating here that in the 23 estimated features, considerable collinearity exists among 5 metrics of jitter and 6 metrics of shimmer (Pearson correlation coefficients ≈ 0.95). Discarding redundancy by selecting one representative metric for jitter and one for shimmer did not improve performance for either classifier.

**Table 2:** Average classification AUC achieved in 100 random iterations using logistic regression (*LR*) and random forest (*RF*) classifiers with Parselmouth (*PM*) feature vectors, mean (*m*) and variance (*v*) feature vectors of 4 types of spectral features, and the combination of these features. ﻿A convolutional neural network (CNN) classifier was also used to classify linear-scale and mel-scale spectrogram images. *LPC:* linear prediction coding, *LAR:* log area ratio, *LPCC:* linear prediction cepstral coefficients, *MFCC:* mel-frequency cepstral coefficients.

		PM	LPC	LPC+PM	LAR	LAR+PM	LPCC	LPCC+MF	MFCC	MFCC+PM	CNN
UAMS dataset	LR	0.60	0.60 (m) 0.66 (v)	0.64 (m) 0.67 (v)	0.56 (m) 0.70 (v)	0.64 (m) 0.67 (v)	0.60 (m) 0.72 (v)	0.67 (m) 0.68 (v)	0.50 (m) 0.73 (v)	0.61 (m) 0.67 (v)	0.97 (mel) 0.95 (linear)
	RF	0.72	0.57 (m) 0.61 (v)	0.66 (m) 0.72 (v)	0.56 (m) 0.66 (v)	0.65 (m) 0.73 (v)	0.56 (m) 0.70 (v)	0.67 (m) 0.77 (v)	0.57 (m) 0.73 (v)	0.68 (m) 0.80 (v)	
mPower dataset	LR	0.70	0.61 (m) 0.62 (v)	0.69 (m) 0.68 (v)	0.61 (m) 0.62 (v)	0.68 (m) 0.68 (v)	0.62 (m) 0.58 (v)	0.68 (m) 0.68 (v)	0.62 (m) 0.60 (v)	0.68 (m) 0.68 (v)	0.94 (mel) 0.92 (linear)
	RF	0.66	0.58 (m) 0.55 (v)	0.66 (m) 0.65 (v)	0.59 (m) 0.58 (m)	0.65 (m) 0.65 (v)	0.57 (m) 0.58 (v)	0.65 (m) 0.66 (v)	0.57 (m) 0.56 (v)	0.64 (m) 0.65 (v)	

### Classification results using spectral features

Four types of spectral feature vectors (see the Methods for details) were estimated in short-time segments using a sliding window. The spectral features include linear prediction coding (LPC) coefficients, log-area ratio (LAR) coefficients, linear prediction cepstral coefficients (LPCC) and mel-frequency cepstral coefficients (MFCC). For each participant, the mean and variance of feature vectors across all segments for the duration of the recording was calculated. Logistic regression and random forest classifiers were applied to the mean and variance spectral feature vectors where each dataset was randomly partitioned into training and testing sets to estimate classification performance and the process was repeated 100 times. For the UAMS dataset, the variance feature vectors (Fig. 1A) outperformed the mean feature vectors (Figure S1A) for all types of spectral features but most notably for LPCC and MFCC as shown in Table 2. Both variance and mean feature vectors performed poorly in the classification task using the mPower dataset (Table 2; Fig. 1B, and Figure S1B). Generally, there was a minor difference in performance between the RF and LR classifiers when applied to the spectral features estimated in each of the two datasets.

### Classification results using combined features

We combined each of the four types of spectral feature vectors (LPC, LAR, LPCC, and MFCC) with the vector of acoustic features estimated using Parselmouth (PM) and examined if the combined features lead to performance gain with the RF or LR classifiers. Since Parselmouth features are more related to the glottal excitation source (vocal folds) and spectral features are more related to tuning effects in the vocal cavity, we hypothesized that a combination of these features may lead to classification performance gain especially using cepstral coefficients (LPCC and MFCC) since glottal excitation and vocal tract spectral components of the speech signal are deconvolved in the cepstral domain^46^. For the UAMS dataset (Fig. 1A), the combination of variance feature vectors of LPCC + PM and MFCC + PM indeed outperformed the separate features using a RF classifier. Using the combination of the mean feature vectors and PM feature vectors did not achieve noticeable gain, with the exception of LR classifier with the UAMS dataset (Figure S1A). Similar advantage to using LPCC + PM or MFCC + PM were not observed for the mPower dataset using either variance (Fig. 1B) or mean (Figure S1B) spectral feature vectors.

**Fig. 2:**
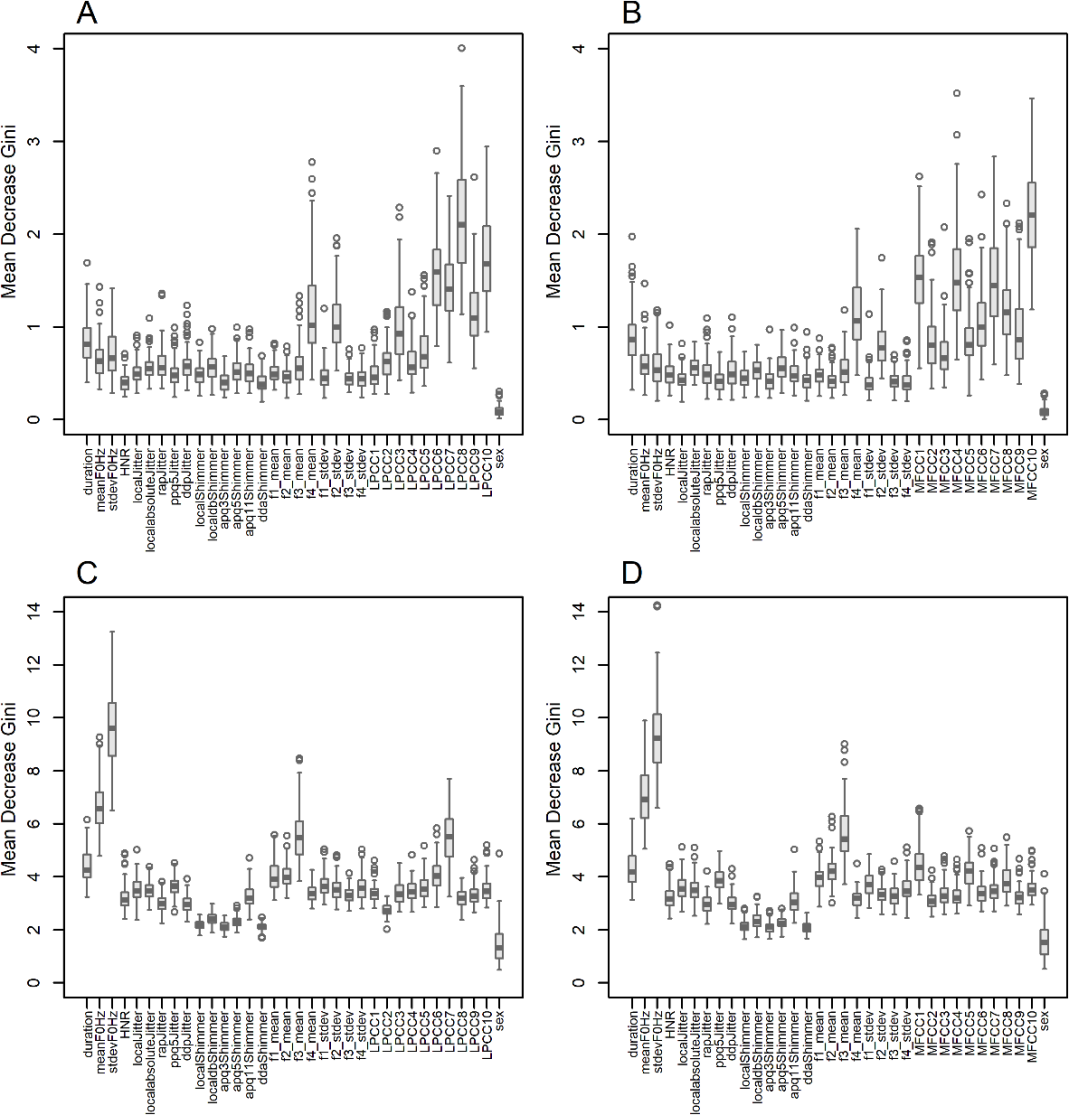
Feature importance of the combined Parselmouth (*PM*) and variance feature vectors of linear prediction cepstral coefficients (*LPCC*) and mel-frequency cepstral coefficients (*MFCC*), assessed by the mean decrease Gini metric of the random forest (*RF*) classifier. (**A**) LPCC + PM for the UAMS dataset, (**B**) MFCC + PM for the UAMS dataset, (**C**) LPCC + PM for the mPower dataset, (**D**) MFCC + PM for the mPower dataset.

Differences in classification performance between the UAMS and mPower datasets can be attributed in part to the differences in feature importance for the classification task. Figure 2 shows feature importance quantified by the mean decrease in Gini values of the RF classifier when the combined variance feature vector of LPCC + PM and MFCC + PM were used with the UAMS and mPower datasets. Most notably, high-order LPCC or MFCC features are most important in the UAMS dataset (Fig. 2A,B) while the standard deviation and mean of the fundamental frequency (F_0_) are the most important features in the mPower dataset (Fig. 2C,D). The difference in the importance of the mean and standard deviation of F_0_ between the UAMS and mPower datasets is also clear when the combined mean cepstral feature vectors and PM feature vectors are used (Figure S2). The same is true when the combination of PM feature vectors and either the mean or the variance feature vectors of LPC or LAR are used (Figures S3 and S4 respectively). We also used R package iml^47^ to estimate the SHapley Additive exPlanation (SHAP) values as described in Štrumbelj et al.^48^. These values estimate feature contribution to prediction decisions made for individual observations. We estimated SHAP feature importance values for the combined PM and variance of MFCC feature vectors with a RF classifier for both the UAMS and mPower datasets. We averaged the absolute SHAP values estimated for all observations used to train the RF classifier in each of the 100 iterations (Figure S5). SHAP values in Figure S5 agreed with the mean decrease in Gini values shown in Fig. 2B,D.

### Classification results with CNN

**Fig. 3:**
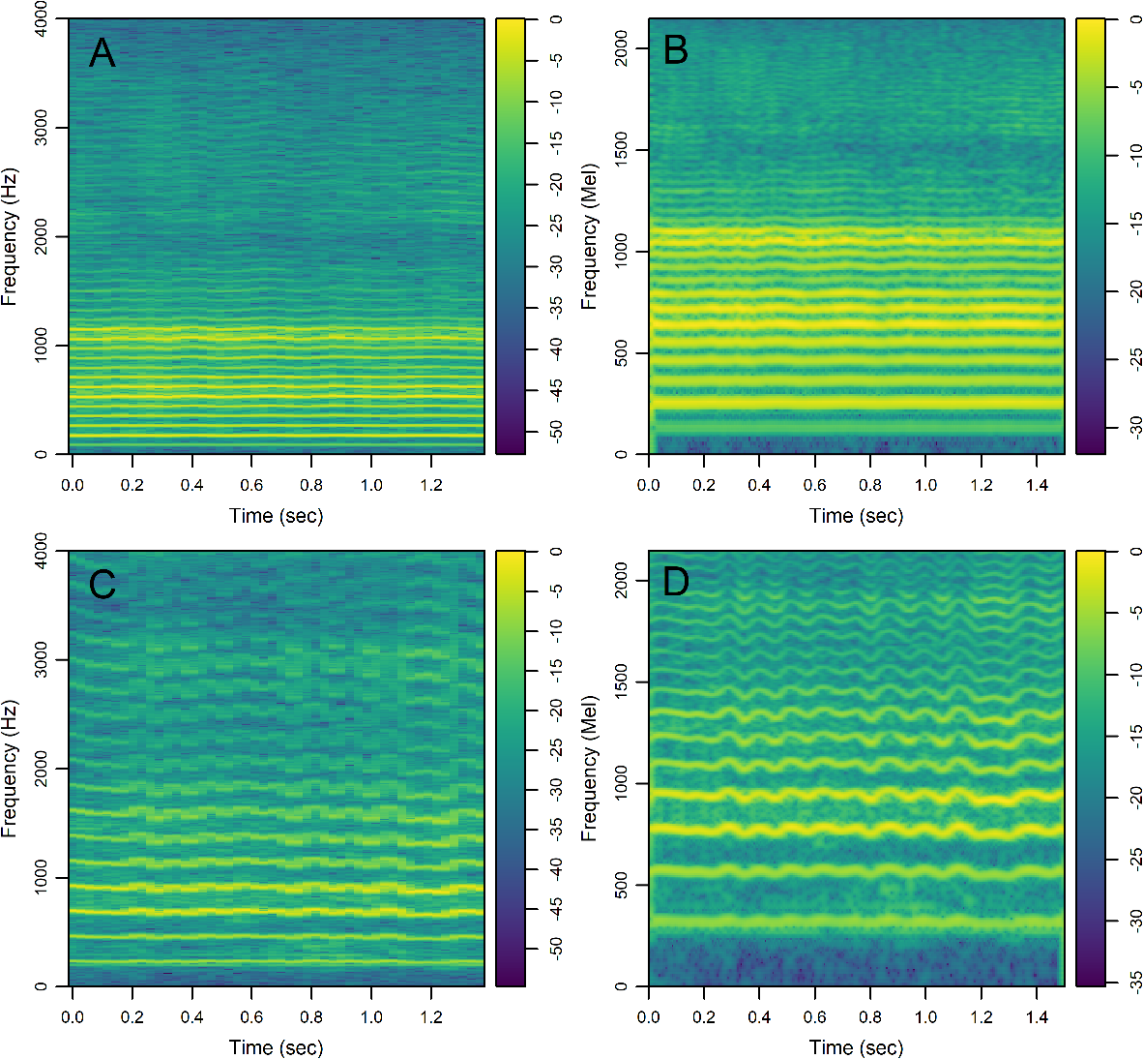
Colored spectrograms of 1.5 s of the sustained vowel /a/ uttered by selected participants. (**A**) Linear-scale spectrogram for a 62 year-old healthy control male, (**B**) mel-scale spectrogram for the same participant in panel A, (**C**) linear-scale spectrogram for a 62 year-old female with Parkinson’s Disease, (**D**) mel-scale spectrogram for the same participant in panel C. Color scale for the linear-scale spectrograms shows 10×log_10_(|S|/max(|S|)), where S represents the complex numbers at the output of the fast Fourier transform. Color scale for the mel-scale spectrograms shows the log-mel spectrogram values normalized by the maximum value.

**Fig. 4:**
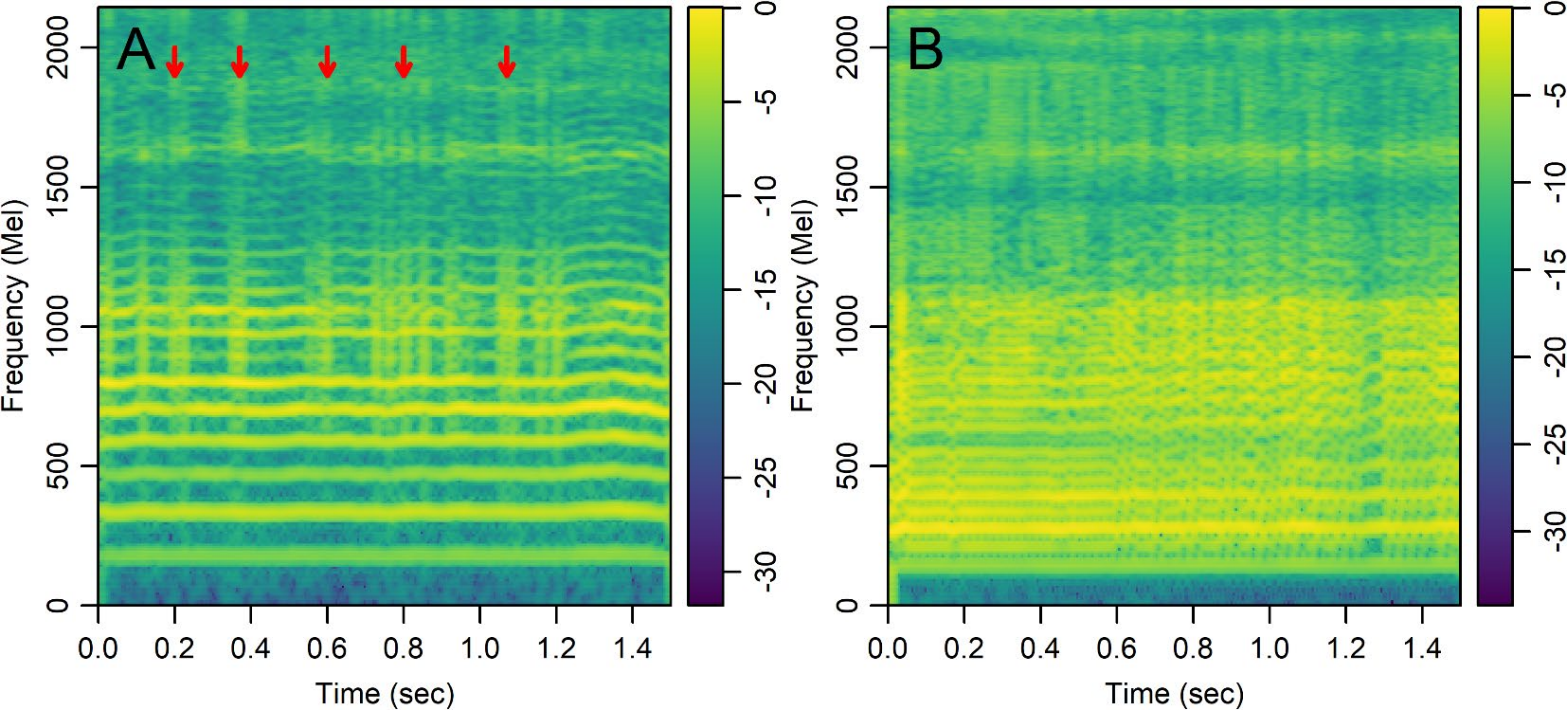
Colored mel-scale spectrograms of 1.5 s of the sustained vowel /a/ uttered by selected people with Parkinson’s disease from the mPower dataset illustrating patterns observed more frequently as compared to healthy controls. (**A**) 57 year-old male showing short subtle distortions in frequency components marked by arrows, (**B**) 69 year-old male showing continuous and severe variations in frequency components. Color scale shows the log-mel spectrogram values normalized by the maximum value.

We analyzed linear-scale and mel-scale spectrogram images (see the Methods for details) of 1.5 s of the sustained vowel /a/ from the UAMS dataset (40 PwPD and 41 HC) and the mPower dataset (188 PwPD and 210 HC). Figure 3 shows sample linear-scale and mel-scale spectrogram images for a 62 year-old healthy control male (Fig. 3A,B) and a 62 year-old female with Parkinson’s disease (Fig. 3C,D) from the mPower dataset. The energy of the voice signal is concentrated around specific frequency components represented by the horizontal bright lines in Fig. 3. Both spectrogram examples show ripples in frequency components across time in the person with PD (Fig. 3C,D) as compared to HC (Fig. 3A,B). This pattern was observed more frequently in spectrograms of PwPD as compared to HC and may indicate lack of control over the fine-tuning of the vocal folds vibration. Other patterns observed more frequently in PwPD as compared to HC were short subtle distortions in frequency components (Fig. 4A, arrows) or continuous and severe frequency variations (Fig. 4B). Since these patterns were not observed in all PwPD, discerning the specific differences in spectrogram images that contribute to classification decisions by the CNN remains a challenge. The classification performance was quantified by the AUC in 100 random iterations, where images were randomly split into 70% training and 30% testing parts in each iteration. The average AUC achieved using linear-scale and mel-scale spectrograms were respectively about 0.95 and 0.97 for the UAMS dataset, and respectively about 0.92 and 0.95 for the mPower dataset (Fig. 1; Table 2). Although the mel-scale spectrograms average AUC performance was only slightly better than linear-scale spectrograms, the difference was statistically significant (Wilcoxon p-value 2.42 × 10^− 5^ and 1.34 × 10^− 10^ for the UAMS and mPower datasets respectively).

In general, the CNN classifier with spectrogram images outperforms both RF and LR classifiers with acoustic and spectral features (Fig. 1; Table 2). Due to the much larger sample size of the mPower dataset (398 samples) as compared to the UAMS dataset (81 samples), the RF and LR classifiers demonstrated smaller variance in AUC values achieved in 100 iterations using the mPower dataset (Fig. 1). The CNN classifier with spectrogram images demonstrated better robustness against heterogeneity between samples by showing similar variance in AUC values in both datasets (Fig. 1).

## Discussion

In this study, we demonstrated the performance of a CNN with transfer learning approach in detecting speech patterns associated with Parkinson’s disease compared to healthy controls in two independently collected datasets. We created and used spectrogram images of the sustained vowel sound /a/ from the mPower dataset^18^ that was collected using a smartphone application. The generated results in this study complement results from an earlier study that demonstrated the same approach with voice recordings collected via telephone lines that support limited bandwidth (UAMS dataset)^40^. The used approach showed excellent classification performance (AUC > 0.9) under both recording environments and outperformed two conventional machine learning classifiers (RF and LR) that used a combination of acoustic and spectral features often used in voice analysis literature. Using 100 random iterations where each dataset is partitioned into 70% training and 30% testing parts, the CNN approach demonstrated better robustness against heterogeneity between samples by having smaller variance in AUC values as compared to RF and LR in both datasets, and achieved comparable AUC variance across the two datasets (Fig. 1). On the other hand, RF and LR classifiers showed more susceptibility to sample size with larger variance in AUC values in the UAMS dataset (81 samples) as compared to the mPower dataset (398 samples). Unlike conventional machine learning methods that require feature vectors, our CNN with transfer learning approach has the advantage of using spectrogram images, allowing it to analyze the speech signal’s energy distribution across time and frequency instead of collapsing features across time as in conventional machine learning methods of voice analysis.

Using the mean decrease Gini metric of the RF classifier to assess feature importance, we found that the most important features are different across the two datasets. The standard deviation and mean of fundamental frequency were the most important features for the mPower dataset, while variance of high-order LPCC or MFCC features were the most important features in the UAMS dataset (Fig. 2). This was validated using SHAP values to assess feature contribution to prediction decisions (Figure S5). In support of the findings reported for the mPower dataset, decreased variation in fundamental frequency was observed in PwPD^49^. Supportive of our reported feature importance in the UAMS dataset, Gillivan-Murphy et al.^50^ showed that PD voice tremor is a vocal tract rather than a purely vocal fold or laryngeal phenomenon (spectral features model the tuning in the vocal tract). In general, the difference in feature importance between two datasets in this study agree with Carron et al.^38^ who found that the most important features for classification vary drastically between two recording environments. They compared the performance of multiple machine learning classifiers using an in-house dataset (captured using professional grade microphones under controlled and supervised settings) and the mPower dataset (captured using smartphones under uncontrolled and unsupervised settings). Although they did not examine telephonic recordings, their results confirm the impact of voice recording platforms on feature importance. Despite the fact that both this study and Carron et al.^38^ used subsets of the mPower dataset, making a direct comparison of most important features is not possible for two reasons: (1) Studies used different feature vectors, and (2) studies used different subsets from the mPower dataset with different sample size (respectively 398 and 60 samples). Additionally, the UAMS dataset was captured using voice messages transferred via telephonic lines that support a limited bandwidth, roughly between 0.3 and 3.4 kHz, resulting in attenuation to the low frequency band that covers fundamental frequencies (F_0_ is typically 100 ~ 146 Hz for healthy males and 188 ~ 221 Hz for healthy females^51^). The estimated average F_0_ of the vowel /a/ using Parselmouth^45^ from the mPower dataset was 116 Hz for healthy males, 123 Hz for males with PD, 192 Hz for healthy females, and 188 Hz for females with PD. The estimated average F_0_ from the UAMS dataset was 112 Hz for healthy males, 152 Hz for males with PD, 194 Hz for healthy females, and 217 Hz for females with PD. These estimates are within the expected range and within the low frequency band that suffers attenuation through telephonic lines. Finding the standard deviation and mean of fundamental frequency to be the most important features in the mPower but not the UAMS dataset can be attributed to this attenuation effect that would only be present in the UAMS dataset.

The mel-scale showed inconsistent advantage when applied to the feature vectors with two conventional machine learning classifiers (RF and LR) resulting in a performance gain only when the MFCC were combined with other acoustic features and used with the RF classifier in the UAMS but not the mPower dataset. However, mel-scale spectrograms outperformed linear-scale spectrograms by a small but statistically significant margin in both mPower and UAMS datasets when used with CNN and transfer learning. Using linear-scale spectrograms of the UAMS dataset, the CNN with transfer learning approach showed marginally lower AUC (AUC = 0.95) as compared to our previous study results (AUC = 0.97)^40^ even though the participant voice samples were the same. In the current study, we used higher frequency resolution spectrograms compared to the previous study. When compared to high resolution, lower frequency resolution results in a blurring effect to the horizontal lines that represent frequency components in spectrogram images. Hires et al.^8^ found small but consistent improvement in classification performance when a Gaussian-blurring kernel was used to smooth pixels and remove extreme outliers in linear-scale spectrogram images. While this suggests that the small difference in our results was due to the different spectrogram resolutions, we cannot completely exclude sampling differences in a small cohort even though the training and testing sets were randomly sampled 100 times for each study. Characterization of the effects of using different image resolutions and/or image blurring methods is beyond the scope of this study.

Spectrogram images showed distinct patterns encountered more frequently in PwPD as compared to HC: (1) Ripples in frequency components indicating perturbation of glottal vibration and inability to sustain stable tones over time (Fig. 3D), (2) short duration distortions in frequency components (Fig. 4A), and (3) continuous and severe variations in frequency components (Fig. 4B). The ripples observed in Fig. 3D may indicate vocal tremors due to the lack of control over the vibration of the vocal folds in PwPD over the duration of the vowel utterance. The spectrograms of PwPD that showed the pattern observed in Fig. 4A had rapid short duration distortions in frequency components occurring aperiodically through the samples and mainly affecting middle and high frequency bands. These distortions may be the visual representation of motor blocks, or freezing in speech that has been described^52–54^, similar to Freezing of Gait, where there is a rapid breakdown in the motor pattern leading to a halt in movement in the feet, or in this case the vocal apparatus. Alternatively, these could be the representation of dystonia due to synchronized inappropriate activation of the muscles in the vocal apparatus. Potentially these could be from the vocal tract rather than the vocal fold or larynx as discussed in Gillivan-Murphy et al. where tremor was not identified in muscles in the vocal folds of PwPD using laryngeal electromyography even when perceived auditorily^50^. The pattern observed in Fig. 4B correlates with low harmonic-to-noise ratio (HNR) values, which indicates increased hoarseness of the voice^55^. Decreased HNR has been reported in PwPD^56,57^, although previous studies^55,58^ found this decrease to be statistically insignificant. This could be explained by the fact that only a small subgroup of PwPD, show a significant decrease in HNR that correlates with the spectrogram pattern seen in Fig. 4B. The different patterns indicated here were encountered in subgroups of all PwPD and discerning the specific differences in spectrogram images that contribute to classification decisions by the CNN with their clinical implications remains a challenge. Other recent studies^21,22^ have examined spectrogram images of PwPD and HC classified using CNN or transformer models and reached contradictory conclusions with respect to the spectrogram regions that are most influential in classification decisions. For example, Malekroodi et al.^21^ showed that spectrogram regions of importance were localized when CNN-based models were used and scattered when transformer-based models were used. The localized regions of importance were different when different CNN-based models were compared. Jeong et al.^22^ showed distinct patterns in a few selected audio recording examples, where high frequency bands in a spectrogram image of a sample incorrectly classified as PwPD (false positive) were most influential for the classification decision while low frequency bands were most influential in a sample incorrectly classified as HC (false negative). These two studies presented patterns in selected examples and refrained from making generalizations regarding the difference between PwPD and HC. The spectrogram images generated in our study showed different irregular patterns in PwPD as compared to HC, confirming the heterogeneity of patterning within PwPD.

Although making a generalizable statement regarding the important spectrogram regions for CNN classification decisions remains a challenge, it is still possible to highlight some common pattern differences between PwPD and HC. We created two average spectrogram images of PwPD and HC using the male group of participants, and the difference between these two images, in the UAMS and mPower datasets separately (Figure S6). Both datasets showed a clear increase in the fundamental frequency in males with PD as compared to HC males. Other narrow frequency bands located roughly between 700 Hz and 1200 Hz (about 780 mel and 1125 mel) were also different, especially in the mPower dataset. Interestingly, these two frequencies are respectively close to the first and second formant frequencies of the vowel sound /a/. The UAMS dataset also showed decreased energy in males with PD at the end of the spectrogram as compared to healthy males. This is likely due to a reduced loudness during the voiced-to-unvoiced transition at the end of the sustained vowel in males with PD. Each spectrogram image was generated from a 1.5 s segment in the middle of each recording. Since the average duration of the mPower and UAMS recordings was respectively 6.8 and 3.3 s, it is likely that spectrograms of the UAMS dataset captured regions adjacent to the transition between voiced and unvoiced parts of the recordings. On the other hand, the mPower recordings were longer and a 1.5 s segment in the middle of each recording would likely exclude transition regions, resulting in more stable loudness.

### Limitations

Although the CNN classifier showed excellent performance in classifying PwPD and HC and specific patterns were observed more frequently in spectrogram images of PwPD as compared to HC, discerning the features or patterns that mostly influence the decisions remain a challenge. While all PwPD in the UAMS dataset were examined by a movement disorders neurologist to make the diagnosis of PD and rule out any other speech, neurologic, or psychiatric confounders, PwPD in the mPower dataset self-reported whether or not they had a diagnosis of PD and no information on how the diagnosis was obtained was provided. All participants in the mPower dataset and HC participants in the UAMS dataset self-reported whether they had any speech, neurologic, or psychiatric disorder and were not examined by a neurologist. Self-reporting might lead to some mislabeled data, which affects the estimated classification performance.

## Conclusion

Convolution neural networks with transfer learning achieve high performance in detecting pathologic speech associated with Parkinson’s disease using spectrogram images of the sustained vowel /a/, with a small but statistically significant gain achieved using mel-scale over linear-scale spectrograms. This approach is equally applicable to voice recorded directly to a smartphone or voice recorded using voice message transferred via telephonic lines with limited bandwidth. This study also shows that recording environments impact the ability of more traditional voice feature analysis to classify pathologic Parkinson’s disease speech. While attributing the classification decisions of the CNN to specific patterns in spectrogram images remains a challenge, distinct patterns were observed in spectrograms of PwPD more frequently as compared to HC. Regardless of this limitation of interpretability, the successful application of the CNN with transfer learning to spectrograms from two different voice recording environments shows the potential of the proposed approach for clinical applications where environments cannot be easily controlled. Future studies may lead to developing a remote monitoring tool for PwPD, including in rural and medically underserved communities where access to technology may still be limited.

## Methods

### Subjects and datasets

The UAMS dataset was collected from 50 PwPD and 50 HC using previously published methods^59^. All voice recordings were collected in compliance with two University of Arkansas for Medical Sciences (UAMS) Institutional Review Board (IRB) approved protocols (UAMS IRB #261021 and #273696) and in compliance with the Declaration of Helsinki. All participants have provided informed consent electronically. PwPD participants received professional diagnoses at the UAMS Movement Disorder Clinic. Demographic data (gender and age) was retrieved from the electronic health records for PwPD and from a RedCap survey for HC participants. Among other tasks, participants were asked to call a secured voicemail number and loudly utter the sustained vowel /a/ for at least 3 s while leaving the voicemail. Each participant contributed one recording. Voice was digitized at 8 kHz sampling frequency where each sample was represented by a 16-bit codeword. Voice recordings in wav file format were made publicly available in a previously published study^40^.

The mPower dataset^18^ was generated using the mPower app which was made available in March 2015 only in the United States for iPhone 4S or newer devices and required iOS8 as a minimum operating system version. Participants were instructed to perform multiple activities including a voice activity where they utter the vowel /a/ into the microphone at a steady volume for up to 10 s. We downloaded the recordings of the sustained vowel /a/ (m4a file format), voice activity and demographic information (csv files) of the mPower dataset from the Synapse database^44^. PwPD and HC subjects were identified respectively as those who answered TRUE and FALSE to receiving a professional PD diagnosis (self-reporting). Subjects who received one or more diagnoses of Depression, Anxiety, Schizophrenia, Bipolar disorder, Asthma, Stroke, or Chronic Obstructive Pulmonary Disease were excluded as these conditions affect voice quality. Subjects self-reported taking PD medication and the time they took medications with respect to when they recorded the voice sample. Therefore, we additionally excluded participants with conflicting record information including PwPD who answered ‘I don’t take Parkinson medications’ and HC subjects who answered anything other than ‘I don’t take Parkinson medications’. Among PwPD who reported taking PD medication, we selected those who reported the medication time-point as ‘Immediately before Parkinson medication’ or ‘Another time’, and excluded those who reported ‘Just after Parkinson medication (at your best)’. We selected subjects in the age range 50–70 year-old. The selection criteria resulted in 229 HC and 215 PwPD subjects. Downloaded recordings of these participants were assessed manually and one good recording per subject was selected when more than one was available. Poor quality recordings such as noisy recordings, recordings in a moving car, recordings in which voices from more than one person were captured, recordings in which bird sounds, or flowing water sound were captured, were excluded. These filtering steps resulted in 210 HC and 188 PwPD recordings to be further processed and analyzed. Supplementary Table S1 provides the record IDs (unique identifiers for recordings) and health codes (unique identifiers for subjects) for the mPower recordings used in this study.

### Data pre-processing

We used the same steps to preprocess the raw recordings of the UAMS dataset as previously published^40^ and saved them in wav file format. All wav and m4a audio files were analyzed using the R environment version 4.1.2^60^. The m4a files of the mPower dataset were converted to wav files using R package *av*^61^. All wav files were imported to the R environment and rescaled to the range [-1,1] using R package *tuneR*^62^. We down-sampled the mPower recordings captured at 44.1 kHz sampling frequency by a factor of 5 to make the recordings as similar as possible to the UAMS dataset and allow the application of the same regression model to both datasets (order depends on sampling frequency). Intervals of silence at the beginning and end of each recording were detected and trimmed when the short-time energy estimated within a sliding window exceeded a threshold level. Any recording shorter than 1.5 s after trimming silent parts was omitted from the analysis, as 1.5 s was deemed the minimum acceptable duration to generate spectrograms. This filtering criteria resulted in 41 HC and 40 PwPD processed recordings from the UAMS dataset, and 210 HC and 188 PwPD recordings from the mPower dataset.

### Acoustic features

Parselmouth^45^ (version 0.4.1), a Python interface to Praat^63^, was used to estimate traditionally studied features associated (code available, see Data Availability Statement) with phonation in sustained vowels including the mean and standard deviation of fundamental frequency (F_0_) and formant frequencies, the harmonics to noise ratio (HNR), and different estimates of jitter and shimmer. Fundamental frequency measures the oscillation rate of the vocal folds in a short segment. Formant frequencies are spectral maxima of the speech waveform that result from the acoustic resonance in the vocal tract. Mean and standard deviations of the first four formants (f_1_, f_2_, f_3_, and f_4_) were included in feature vectors. The standard deviations of F_0_ and formant frequencies provide an assessment of the ability of a speaker to sustain stable tones across time. HNR is the ratio of periodic to non-periodic components of the speech segment. Jitter describes the fundamental frequency variation over time, and shimmer describes the variation in signal amplitude over time. The features were estimated using Parselmouth with default parameter values over the duration of the sustained vowel /a/. A total of 23 features were used as a feature vector for the classification task similar to our previously published study^40^. One feature vector was generated per participant (subject), ensuring the independence of identities between the training and testing sets when the classifiers are trained.

### Spectral features

Spectral feature vectors were estimated using the methods from our previously published study^40^. Briefly, speech was analyzed in a sliding window of 256 samples or 32 milliseconds (msec) with 50% overlap. Processed recordings from the UAMS and mPower datasets had respectively an average duration of 3.3 and 6.8 s. On average, features were estimated in 205 windows for the UAMS dataset and 424 windows for the mPower dataset. Within each window, speech signal was fitted to an autoregressive model of order *p* = 10 using R package *gsignal*^64^ and the Levinson-Durbin algorithm^65^ was used to solve the resulting Yule-Walker equations. The solution generated the LPC coefficients and the partial correlation coefficients that were converted to the LAR feature vector. LPCC were generated using a recursion approach from R package *tuneR*^62^. MFCC where estimated using R package *tuneR*^62^. Cepstral coefficients deconvolve the glottal excitation source and the vocal tract spectral components of the speech signal^46^. Mel-frequency scale models the perceptual frequency response of the human ear which is approximately linear below 1 kHz and logarithmic above 1 kHz. The mean and variance of each of the 10 estimated coefficients of the four types of features (LPC, LAR, LPCC, and MFCC) were calculated and used as input mean or variance feature vectors for logistic regression and random forest classifiers. Having one mean and one variance spectral features vector per participant (subject) ensures the independence of identities between the training and testing sets when the classifiers are trained.

### Machine learning classifiers

LR and RF classifiers were used to assess the classification performance of acoustic features, spectral features, and both combined, separately for each dataset. A generalized logistic regression model with forward stepwise feature selection and 3-fold cross-validation was trained using the R package *caret*^66^ and the best model was selected based on the Akaike information criterion (AIC) from the R package *MASS*^67^. We note that the best models in different iterations mostly satisfied the model assumptions of having a binary outcome, low multicollinearity between predictors, and no influential values in the predictors that affect the model. However, we acknowledge that some predictor variables showed linear association with the outcome while others did not which may affect the accuracy of the model in predicting outcome. Breiman’s algorithm^68^ was used to build the RF model as implemented in R package *randomForest*^69^ (number of trees = 1000, randomly sampled candidate variables at each split = 6, terminal nodes minimum size = 5). We used a larger number of trees in our RF classifier than the minimum needed to achieve similar performance at the cost of a negligible increase in computational complexity. No overfitting was observed based on the average difference between the AUC values estimated from the training and testing (out-of-bag) parts. To improve the robustness of performance evaluation, repeated cross-validation was performed by randomly splitting each dataset into 70% training and 30% testing parts. Splitting was repeated 100 times and the Area Under the receiver operating characteristic Curve (AUC) was estimated in each iteration (Fig. 1). The variability in estimated AUC values over iterations provides an assessment of classifier robustness against random splits. Importance of individual features was assessed using the mean decrease Gini metric estimated by the RF classifier (Fig. 2). The higher the value of the mean decrease Gini score, the higher the importance of the variable in the classifier model.

### Spectrograms

We created spectrogram images of the sustained vowel /a/ for both PwPD and HC recordings for the classification with CNN task. To make all images directly comparable, all recordings were trimmed such that only 1.5 s is used to generate spectrograms. Processed recordings from the UAMS dataset had an average duration of 3.3 s, while processed recordings from the mPower dataset lasted longer with an average duration of 6.8 s. Many participants in the mPower dataset could not sustain the vowel /a/ for the full duration and instead repeated it twice or more. An in-house R code (code available, see Data Availability Statement) was used to clip silent segments and select the longest continuous voiced segment when there were more than one. Linear-scale spectrogram data was generated using function *specgram* from R package g*signal*^64^ with Hanning sliding window of 1024 samples (128 msec), 75% overlap rate, and 1024 fast Fourier transform (FFT) size. These parameters resulted in 48 time windows or segments per spectrogram image. In our previously published study^40^, we used lower frequency resolution with Hanning sliding window of 256 samples (32 msec) and 50% overlap rate. Spectrogram images show the distribution of speech waveform energy across time and frequency axes using color intensities. The color scale shows 10×log_10_(|S|/max(|S|)), where S represents the complex numbers at the output of the FFT (Fig. 3). Images were created using function *imagep* from R package *oce*^70^ and saved in jpg file format with 600 × 600 pixels, and 24-bit color depth. Mel-scale spectrograms were generated using R package *torchaudio*^71^ with Hanning sliding window of 512 samples (58 msec), 90% overlap rate, 1024 FFT size, and 256 mel filter banks. These parameters resulted in 260 time windows per spectrogram image. Images were created using function *imagep* from R package *oce*^70^ and saved in jpg file format with similar parameters to linear-scale spectrograms. The color scale shows log-mel spectrogram values normalized by the maximum value. Parameters used to generate both linear-scale and mel-scale spectrograms were selected to achieve a compromise between time resolution and frequency resolution based on visual inspection of generated images with different combinations of parameters. One linear-scale and one mel-scale spectrogram image were generated per participant, ensuring the independence of identities between the training and testing sets when the CNN model is trained. This allows the CNN model to capture information associated with the PD status rather than with individuals which leads to inflated model performance^72^.

### Convolutional neural network (CNN)

Similar to our previous study^40^, we applied the Inception V3 CNN architecture^73^ pre-trained on the ImageNet database^12^ to classify spectrogram images. The Inception V3 architecture has shown successful adaptation to medical imaging problems through transfer learning^74,75^. The pre-trained model has a chain of digital filters with parameters tuned to extract meaningful features that enable the CNN to solve image classification problems. Our datasets were used to perform additional training to adapt that ability to the specific problem of classifying spectrogram images into HC and PwPD phenotypes using transfer learning. The original classification stage of the Inception model was replaced with four custom layers: batch normalization, 2 dense layers (1024 nodes, relu activation) and a final dense layer (2 classes, softmax activation) to create a multi-layer perceptron (MLP) classifier stage.

Batch normalization was applied prior to the MLP classifier to minimize internal covariate shift and reduce the number of training iterations required. Batch normalization was developed to allow higher learning rates and faster convergence^76^. It has been shown that applying batch normalization followed by dropout serves to pre-whiten the data, improving training performance of MLPs^77^. We employed this combination to enable adequate training of our MLP classifier with limited data.

For the classification task, we analyzed linear-scale and mel-scale spectrogram images of the sustained vowel /a/ in each dataset (UAMS and mPower) separately. Images were normalized to the range [0,1] and randomly split into 70% training and 30% testing parts. The random split was repeated 100 times and the AUC, accuracy, and loss were estimated in each iteration. This approach has been described in the literature as repeated holdout or repeated cross-validation^78^. Repeated cross-validation is more suitable for small datasets with no independent test set^79^. It maximizes data utilization by using all samples for training and testing, and it reduces variance in performance estimates compared to a single data split with holdout. We performed repeated cross-validation to improve the robustness of the AUC evaluation. Figure 1 shows boxplots of the estimated AUC values from the 100 iterations. The small inter-quartile range of the estimates using linear-scale or mel-scale spectrogram images from both the mPower and UAMS datasets demonstrate the robustness of the CNN classifier against specific data splits for both relatively small and medium size datasets. The effect of dataset size on the inter-quartile range of the AUC estimates was clearer when the random forest and logistic regression classifiers were used.

To minimize the problem of overfitting only the classifier is trained using our data. We use a small number of epochs (10), a batch size of 4, a dropout of 20%, and the adam optimizer with an initial learning rate of 0.001 to adjust the learning rate. Image augmentation was not applied. The validation loss continues to decrease for all four test cases (UAMS and mPower data, linear-scale and mel-scale spectrograms) and tracks the training loss indication the model does not overfit the data.

The Software implementation of our pre-trained CNN approach is available as an open source Jupyter notebook on github to provide additional transparency and clarity regarding the implementation. All CNN-based analysis was performed on a MacBook Pro with a 10 core M1 processor and 32 GB of memory.

## Electronic supplementary material

Below is the link to the electronic supplementary material.

Supplementary Material 1

Supplementary Material 2

## Data Availability

Participant voice recordings for data from the University of Arkansas for Medical Sciences are available from figshare as “Voice Samples for Patients with Parkinson’s Disease and Healthy controls”, https://doi.org/10.6084/m9.figshare.23849127. Institutional IRB and regulatory affairs decisions equate the spectrogram images created from these files to a voice print which is protected health information and cannot be publicly shared. Figures 3 and 4 are non-computable illustrations of these data and publication is permitted by the same institutional authorities. Data from the mPower study are available from https://www.synapse.org/Synapse: syn4993293/wiki/247860. Software implementation of the CNN algorithm, the code used to extract acoustic features using Parselmouth, and R codes to generate mel-scale and linear scale spectrogram images from audio files of the UAMS and mPower datasets are available on https://github.com/uams-tri/PD-Voice under an Apache 2.0 license. The CNN and Parselmouth codes were written in python and presented as Jupyter notebooks. The associated environment configuration YAML file for the CNN algorithm is also provided.
