# Supplementary material for: Pre-trained convolutional neural networks identify Parkinson’s disease from spectrogram images of voice samples
Source: Sci Rep. 2025 Mar 1;15:7337. doi: 10.1038/s41598-025-92105-6 (PMC11873116; doi:10.1038/s41598-025-92105-6)
Supplement: Supplementary file 1 [file 41598_2025_92105_MOESM1_ESM.pdf]

Pre-trained Convolutional Neural Networks Identify Parkinson's Disease from Spectrogram Images of Voice Samples (Additional File 1 - Supplementary Figures)

Yasir Rahmatallah[1], Aaron Kemp[1], Anu Iyer[2], Lakshmi Pillai[3],
Linda Larson-Prior[1,3,4], Tuhin Virmani[1,3], and Fred Prior[1]

[1] Department of Biomedical Informatics,
University of Arkansas for Medical Sciences,
Little Rock, AR 72205, USA
[2] Georgia Institute of Technology, Atlanta, GA 30332, USA
[3] Department of Neurology,
University of Arkansas for Medical Sciences,
Little Rock, AR 72205, USA
[4] Department of Neuroscience,
University of Arkansas for Medical Sciences,
Little Rock, AR 72205, USA

January 8, 2025

List of Figures

S1 Estimated classification performance metric quantified by the area under the receiver operating characteristic curve (AUC) achieved in 100 iterations using random forest (RF) and logistic regression (LR) classifiers with the Parselmouth (PM) and *mean* feature vectors of four types of spectral features (separately and combined) and using the pre-trained convolutional neural network (CNN) with mel-scale and linear-scale spectrogram images. A) Results from the UAMS dataset, B) results from the mPower dataset. LPC: linear prediction coding, LAR: log-area ratio, LPCC: linear prediction cepstral coefficients, MFCC: mel-frequency cepstral coefficients. 3

S2 Feature importance of the combined Parselmouth (PM) and *mean* feature vectors of linear prediction cepstral coefficients (LPCC) and mel-frequency cepstral coefficients (MFCC), assessed by the mean decrease Gini metric of the random forest (RF) classifier. A) LPCC+PM for the UAMS dataset, B) MFCC+PM for the UAMS dataset, C) LPCC+PM for the mPower dataset, D) MFCC+PM for the mPower dataset. 4

S3 Feature importance of the combined Parselmouth (PM) and *variance* feature vectors of linear prediction coding (LPC) coefficients and log-area ratio (LAR) coefficients, assessed by the mean decrease Gini metric of the random forest (RF) classifier. A) LPC+PM for the UAMS dataset, B) LAR+PM for the UAMS dataset, C) LPC+PM for the mPower dataset, D) LAR+PM for the mPower dataset. 5

S4 Feature importance of the combined Parselmouth (PM) and *mean* feature vectors of linear prediction coding (LPC) coefficients and log-area ratio (LAR) coefficients, assessed by the mean decrease Gini metric of the random forest (RF) classifier. A) LPC+PM for the UAMS dataset, B) LAR+PM for the UAMS dataset, C) LPC+PM for the mPower dataset, D) LAR+PM for the mPower dataset. 6

S5 Feature importance of the combined Parselmouth (PM) and *variance* feature vectors of mel-frequency cepstral coefficients (MFCC) when used with the random forest (RF) classifier, assessed by the Shapley additive explanation (SHAP) values. A) UAMS dataset, B) mPower dataset. 7

S6 Difference between the average of spectrogram images of healthy controls (HC) and people with Parkinson's disease (PwPD) in the male group of the UAMS dataset (panel A) and male group of the mPower dataset (panel B). Light color indicates larger energy for HC and dark color indicates larger energy for PwPD. Both datasets show increased fundamental frequency for PwPD as compared to HC. Each spectrogram image was generated from 1.5 seconds segment in the middle of each recording after trimming silence. Since the average duration of the mPower and UAMS recordings was respectively 6.8 and 3.3 seconds, it is likely that spectrograms of the UAMS dataset captured adjacent regions to the transition between the voiced and unvoiced parts of the recordings. The UAMS dataset shows decreased energy at the end of the spectrogram (around time=1.4 seconds) for PwPD as compared to HC, likely due to a reduced loudness during the voiced-to-unvoiced transition at the end of the sustained vowel in males with Parkinson's disease. 8

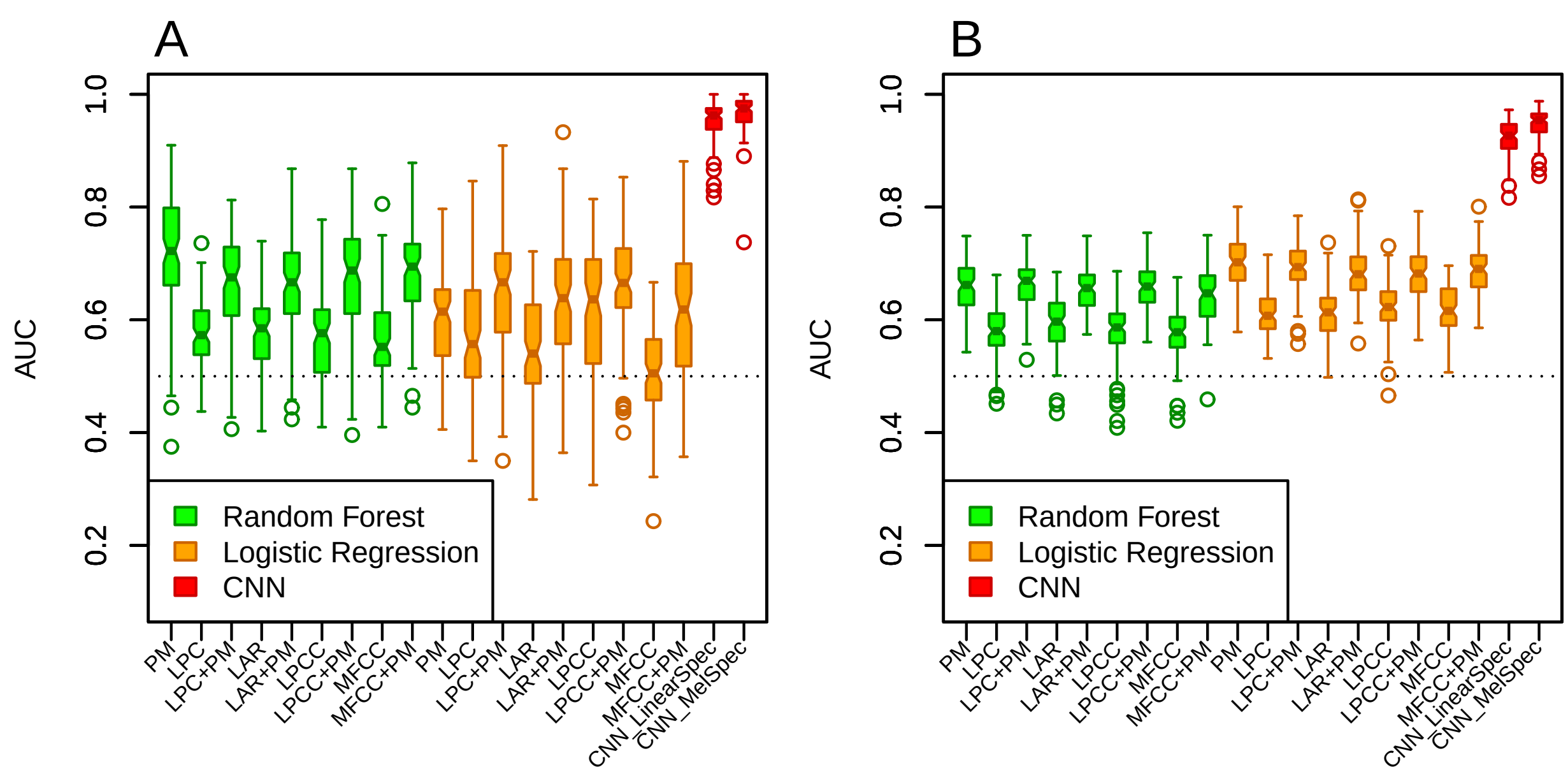

Figure S1: Estimated classification performance metric quantified by the area under the receiver operating characteristic curve (AUC) achieved in 100 iterations using random forest (RF) and logistic regression (LR) classifiers with the Parselmouth (PM) and *mean* feature vectors of four types of spectral features (separately and combined) and using the pre-trained convolutional neural network (CNN) with mel-scale and linear-scale spectrogram images. A) Results from the UAMS dataset, B) results from the mPower dataset. LPC: linear prediction coding, LAR: log-area ratio, LPCC: linear prediction cepstral coefficients, MFCC: mel-frequency cepstral coefficients.

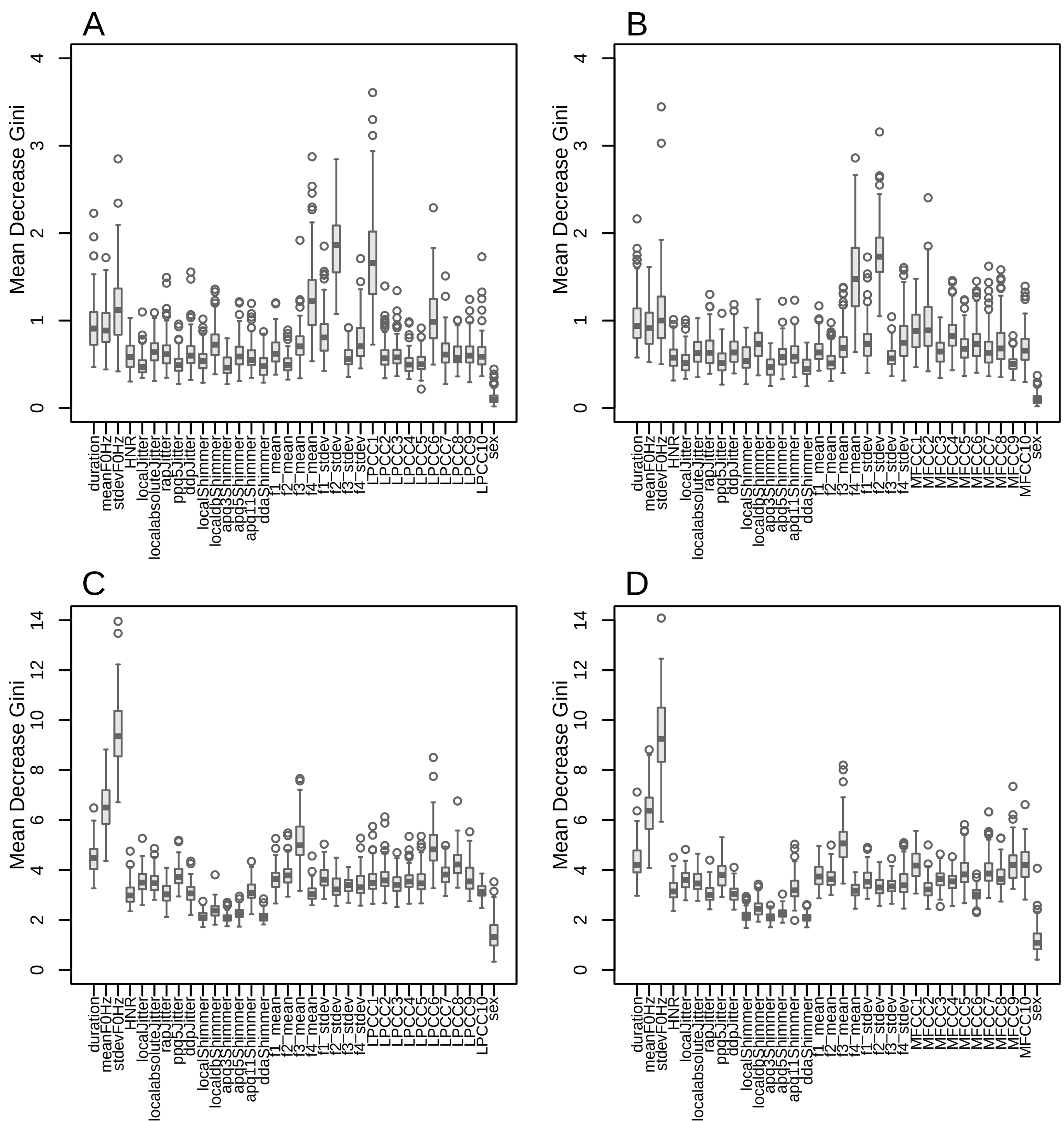

Figure S2: Feature importance of the combined Parselmouth (PM) and *mean* feature vectors of linear prediction cepstral coefficients (LPCC) and mel-frequency cepstral coefficients (MFCC), assessed by the mean decrease Gini metric of the random forest (RF) classifier. A) LPCC+PM for the UAMS dataset, B) MFCC+PM for the UAMS dataset, C) LPCC+PM for the mPower dataset, D) MFCC+PM for the mPower dataset.

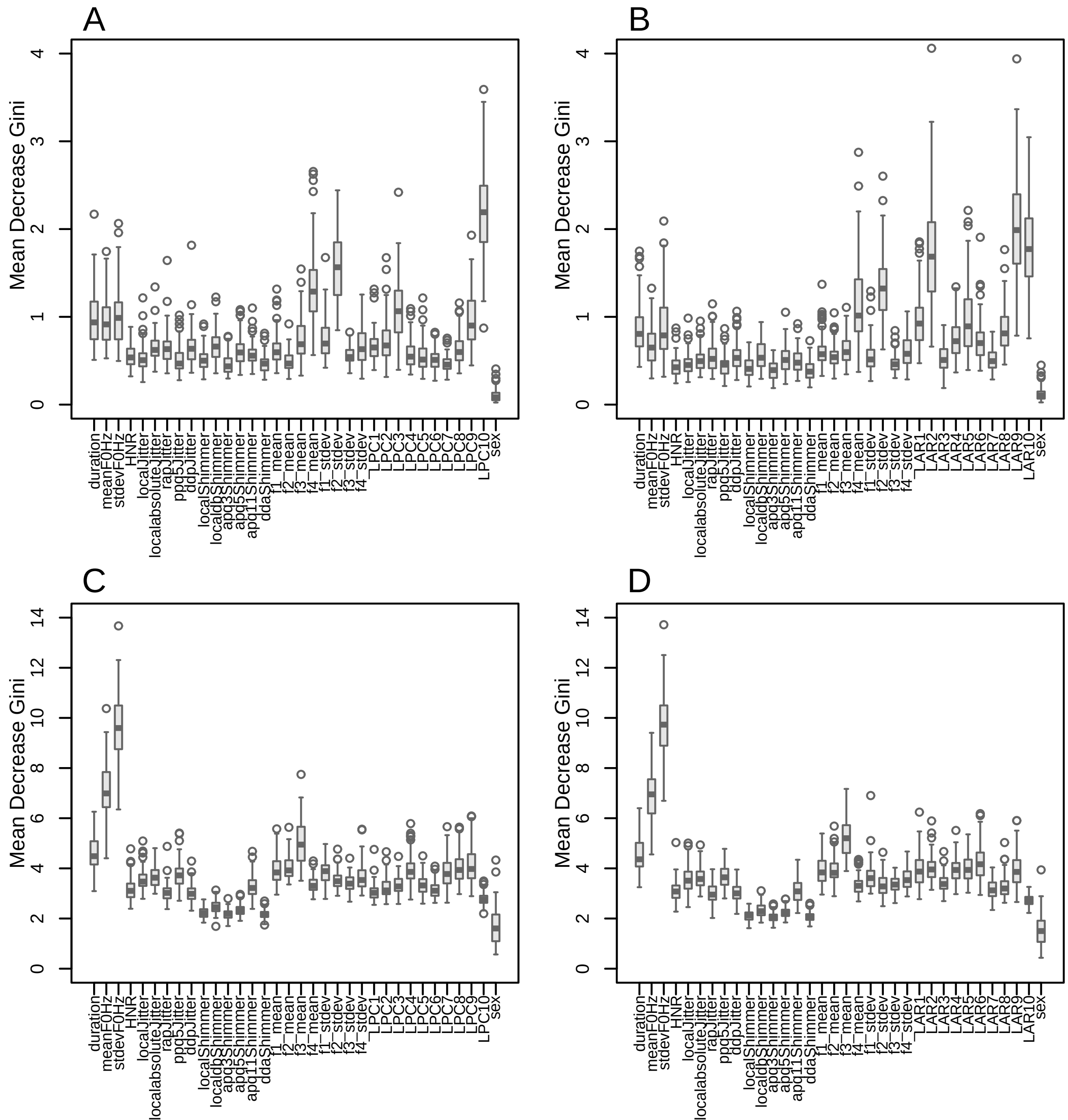

Figure S3: Feature importance of the combined Parselmouth (PM) and *variance* feature vectors of linear prediction coding (LPC) coefficients and log-area ratio (LAR) coefficients, assessed by the mean decrease Gini metric of the random forest (RF) classifier. A) LPC+PM for the UAMS dataset, B) LAR+PM for the UAMS dataset, C) LPC+PM for the mPower dataset, D) LAR+PM for the mPower dataset.

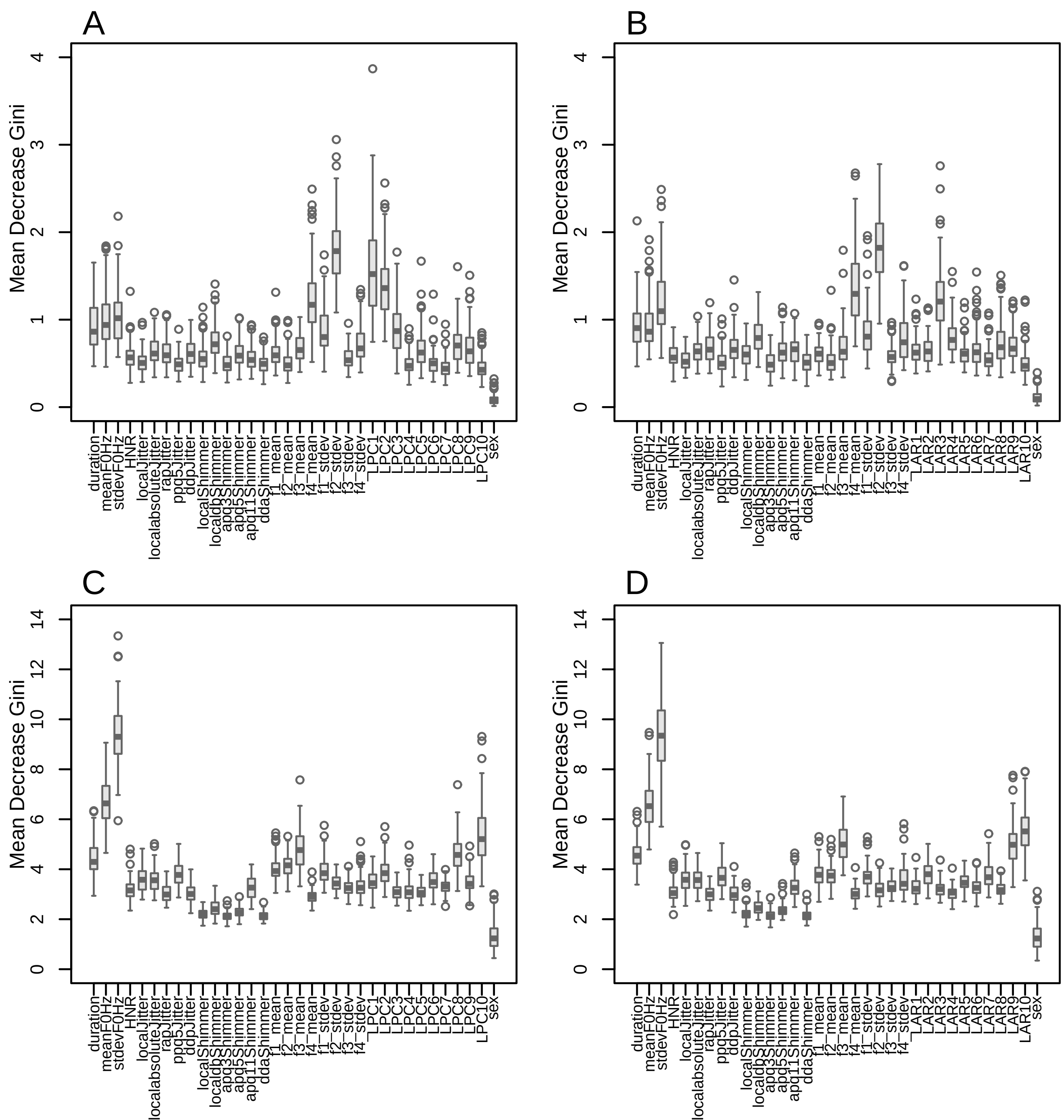

Figure S4: Feature importance of the combined Parselmouth (PM) and *mean* feature vectors of linear prediction coding (LPC) coefficients and log-area ratio (LAR) coefficients, assessed by the mean decrease Gini metric of the random forest (RF) classifier. A) LPC+PM for the UAMS dataset, B) LAR+PM for the UAMS dataset, C) LPC+PM for the mPower dataset, D) LAR+PM for the mPower dataset.

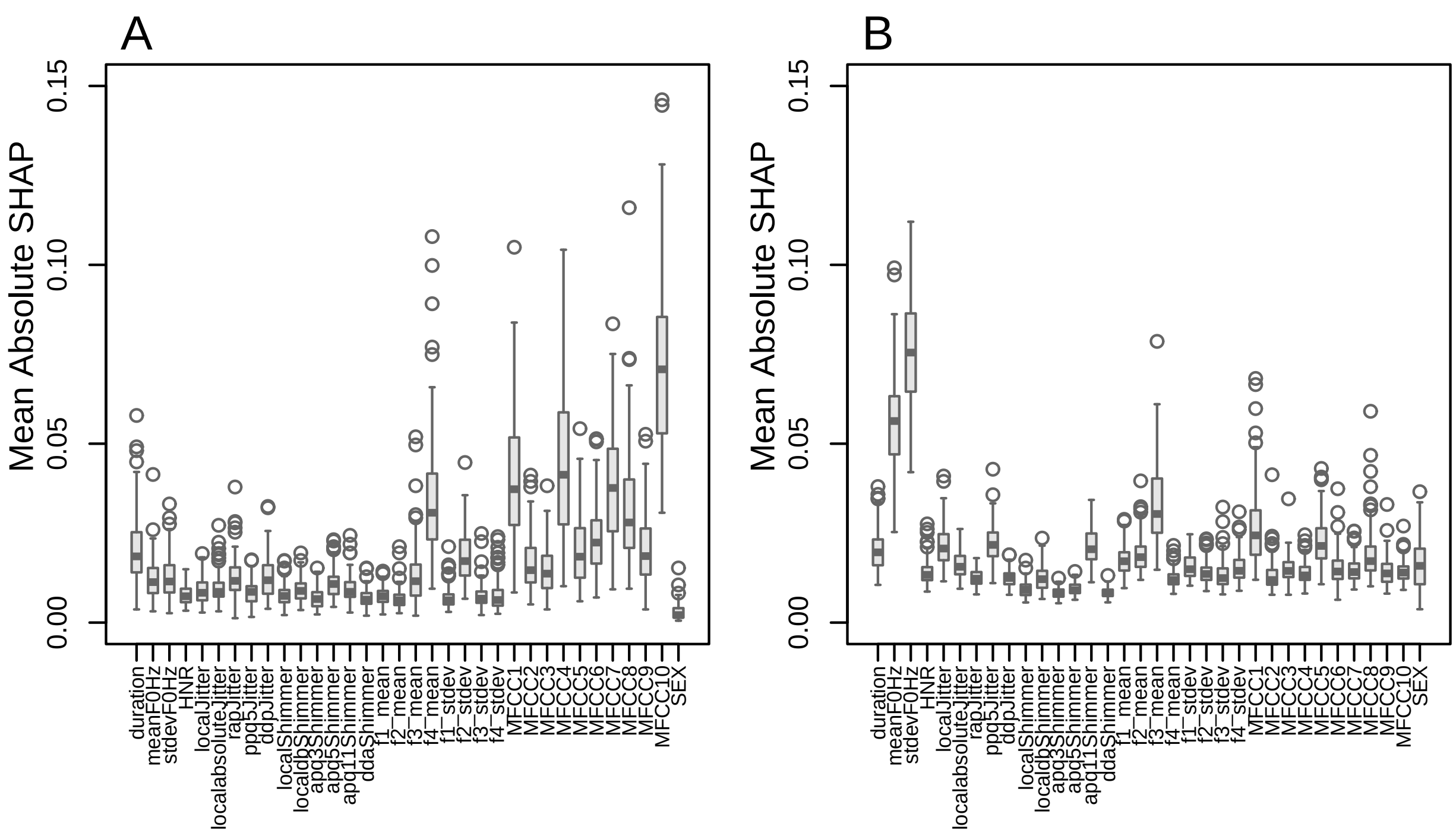

Figure S5: Feature importance of the combined Parselmouth (PM) and *variance* feature vectors of mel-frequency cepstral coefficients (MFCC) when used with the random forest (RF) classifier, assessed by the Shapley additive explanation (SHAP) values. A) UAMS dataset, B) mPower dataset.

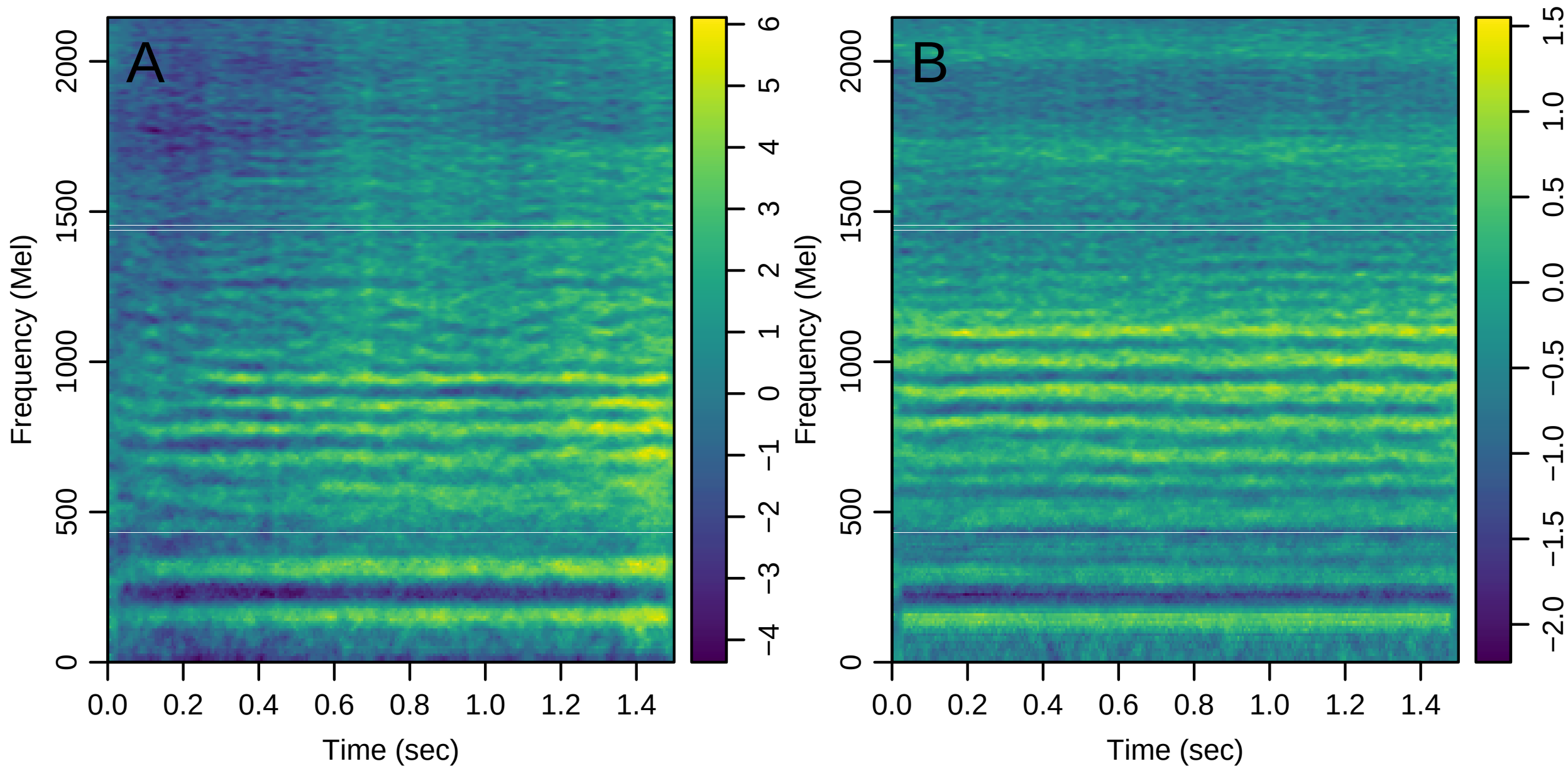

Figure S6: Difference between the average of spectrogram images of healthy controls (HC) and people with Parkinson's disease (PwPD) in the male group of the UAMS dataset (panel A) and male group of the mPower dataset (panel B). Light color indicates larger energy for HC and dark color indicates larger energy for PwPD. Both datasets show increased fundamental frequency for PwPD as compared to HC. Each spectrogram image was generated from 1.5 seconds segment in the middle of each recording after trimming silence. Since the average duration of the mPower and UAMS recordings was respectively 6.8 and 3.3 seconds, it is likely that spectrograms of the UAMS dataset captured adjacent regions to the transition between the voiced and unvoiced parts of the recordings. The UAMS dataset shows decreased energy at the end of the spectrogram (around time=1.4 seconds) for PwPD as compared to HC, likely due to a reduced loudness during the voiced-to-unvoiced transition at the end of the sustained vowel in males with Parkinson's disease.
